# Redox–Genomic Crosstalk: Linking Oxidative Stress, Sperm DNA Fragmentation, and Epigenetics in Personalized Management of Male Infertility

**DOI:** 10.3390/jpm16020079

**Published:** 2026-02-01

**Authors:** Pallav Sengupta, Sulagna Dutta, Mohamed AlaaEldein Elsuity, Ramadan Saleh

**Affiliations:** 1 Department of Biomedical Sciences, College of Medicine, Gulf Medical University, Ajman P.O. Box 4184, United Arab Emirates; 2 Basic Medical Sciences Department, College of Medicine, Ajman University, Ajman P.O. Box 346, United Arab Emirates; 3 Center of Medical and Bio-Allied Health Sciences Research, Ajman University, Ajman P.O. Box 346, United Arab Emirates; 4 Department of Dermatology, Venereology and Andrology, Faculty of Medicine, Sohag University, Sohag 82524, Egypt; mohammed.suity@gmail.com; 5 Ajyal IVF Center, Ajyal Hospital, Sohag 82511, Egypt

**Keywords:** epigenetics, male infertility, oxidative stress, sperm DNA fragmentation

## Abstract

Male infertility is increasingly recognized as a complex, multifactorial disorder that extends beyond abnormalities in conventional semen parameters. A growing body of evidence highlights oxidative stress, sperm DNA fragmentation (SDF), and epigenetic alterations as tightly interconnected mechanisms contributing to sperm dysfunction and impaired fertility. Reactive oxygen species, though vital for sperm maturation and signaling, can inflict extensive genomic and chromatin damage when their levels exceed the antioxidant capacity of the testis and seminal plasma. These redox-driven lesions not only compromise fertilization potential but may also influence embryonic development and offspring health. Clinical studies and meta-analyses consistently report that elevated SDF and redox imbalance are associated with reduced pregnancy and live birth rates, particularly in assisted reproductive technologies (ARTs). The use of testicular sperm in men with high ejaculated SDF appears to improve ART outcomes, although long-term safety data remain limited. Advances in redox and genomic diagnostics, including assays for oxidation–reduction potential, SDF, and sperm epigenetic profiling, have opened new avenues for precision-based andrology, enabling targeted antioxidant, metabolic, and surgical interventions. Nonetheless, methodological variability, lack of assay standardization, and insufficient longitudinal follow-up constrain the full clinical translation of these findings. This review synthesizes evidence linking OS, SDF, and epigenetic alterations, highlighting their mechanistic crosstalk and translational relevance in the personalized management of male infertility.

## 1. Introduction

Male infertility remains a major contributor to the global burden of reproductive failure, accounting for nearly half of all infertility cases [1]. However, a substantial proportion of men with normal semen parameters continue to experience infertility and a diagnostic gray zone persists despite advances in molecular andrological testing [2]. This residual category, often termed ‘unexplained’, reflects an incomplete understanding of the molecular architecture that governs sperm function beyond conventional semen analysis [3]. Over the past decade, oxidative stress (OS) and genomic instability have emerged as critical, intersecting determinants of sperm dysfunction [4]. Reactive oxygen species (ROS), while indispensable for physiological processes such as capacitation and acrosome reaction, can rapidly shift from signaling mediators to cytotoxic agents under conditions of redox imbalance and OS, causing structural and functional damage to sperm DNA [5]. The consequences of OS are not confined to fragmentation of sperm DNA alone [6], rather ROS-induced damage can initiate epigenetic reprogramming, altering DNA methylation, histone retention, and small RNA signatures, with potential transgenerational repercussions on embryonic development and offspring health [7]. This interconnected redox–genomic–epigenomic network should be viewed as a continuous and adaptive system, not a collection of separate processes. It offers a compelling biological explanation for the observed variability in reproductive success among men with comparable semen characteristics, particularly in the context of assisted reproductive technologies (ART) [8].

Current clinical frameworks occasionally integrate redox and genomic diagnostics into male infertility workups [9]. Despite growing use of advanced semen tests, a large proportion of male infertility remains classified as ‘idiopathic’, reflecting a gap between molecular insights and clinical decision-making. The absence of an integrated framework linking oxidative stress, genomic instability, and epigenetic dysregulation limits diagnostic precision and rational therapy selection. Addressing this gap is critical for advancing personalized andrology and improving reproductive outcomes. As a result, therapeutic decisions remain largely experiential, with antioxidant supplementation used indiscriminately and often without biomarker guidance. A paradigm shift is therefore necessary, from ‘one-size-fits-all’ empiricism toward phenotype-driven, redox–genomic endotyping. Accordingly, this review aims to: (i) integrate oxidative, genomic, and epigenetic mechanisms into a unified redox–genomic framework; (ii) define clinically actionable redox–genomic endotypes of male infertility; and (iii) translate these mechanisms into practical diagnostic and therapeutic strategies for personalized management.

To meet these aims, a narrative literature search was conducted using PubMed, Scopus, and Web of Science, focusing on studies published primarily over the last 15 years. Keywords included oxidative stress, sperm DNA fragmentation, epigenetics, redox biology, and male infertility. Priority was given to mechanistic studies, clinical investigations, meta-analyses, and translational evidence relevant to personalized andrology.

## 2. Novelty and Significance of the Present Review

The relationship between OS, SDF, and epigenetic alterations in male infertility has been extensively reviewed, with contributions ranging from foundational mechanistic descriptions to recent publications addressing specific aspects of this triad [10,11,12,13,14,15]. We acknowledge these contributions and clarify the distinctive features of our manuscript.

First, while existing reviews predominantly focus on isolated aspects (namely OS mechanisms, antioxidant therapeutics, microRNA regulation, or transgenerational epigenetic inheritance), our review uniquely conceptualizes the “redox–genomic–epigenomic crosstalk” as an integrated, self-reinforcing axis. We detail bidirectional mechanistic loops, such as how 8-OHdG accumulation inhibits DNA methyltransferase binding, creating hypomethylation that renders chromatin more susceptible to further oxidative cleavage. This systems-level integration explains why addressing single components often yields inconsistent clinical outcomes.

Second, we introduce five clinically actionable “redox–genomic endotypes”: (i) Oxidative-Dominant Infertility, (ii) Redox-Driven DNA Fragmentation, (iii) Chromatin/Epigenetic Dysfunction with Redox Overlay, (iv) Epigenetic-Dominant Endotype, and (v) SDF-Isolated Endotype. No existing publication offers such phenotype-stratified classification linking molecular signatures to tailored therapeutic strategies, transforming “idiopathic” male infertility management from empirical trial-and-error to mechanism-guided intervention.

Third, we provide comprehensive analysis of genetic polymorphisms modulating oxidative susceptibility and repair capacity, including SOD2, GPx1, CAT, GSTM1/GSTT1, OGG1, XRCC1, and NOS3 variants. No prior review systematically consolidates these into a coherent “genetic oxidative haplotype” framework or proposes genotype-guided antioxidant selection, a pharmacogenomic dimension largely unexplored in reproductive medicine.

Fourth, we address ancestry-specific reference intervals for oxidative and epigenetic biomarkers, recognizing that baseline values and allele frequencies vary across ethnic and dietary contexts a precision medicine consideration overlooked in reviews assuming universal thresholds.

Fifth, we synthesize meta-analytical evidence on SDF impact on ART outcomes and critically address offspring safety considerations. These include associations between paternal SDF and miscarriage rates or transgenerational effects, thereby balancing efficacy with long-term safety.

Finally, we emphasize clinical translation through practical guidance on measurement protocols, comparative assay evaluation, and diagnostic integration, bridging the gap between bench research and bedside implementation that persists in mechanistically focused literature.

## 3. Redox Biology Across Spermatogenesis

The process of spermatogenesis integrates proper orchestration of mitotic, meiotic, and post-meiotic differentiation that transforms diploid spermatogonia into highly specialized spermatozoa [16]. The testicular microenvironment is dynamically regulated by redox status, which determines whether ROS serve as essential signaling molecules or potential cytotoxins [17]. Optimal levels of ROS modulate germ cell proliferation, chromatin remodeling, and capacitation [5]. However, excessive accumulation leads to OS, impairing spermatogenic efficiency and genomic integrity. The testicular milieu, therefore, maintains a fine oxidative-antioxidative equilibrium critical for sperm functional competence [18].

### 3.1. Endogenous and Exogenous ROS Sources

**Figure 1:**
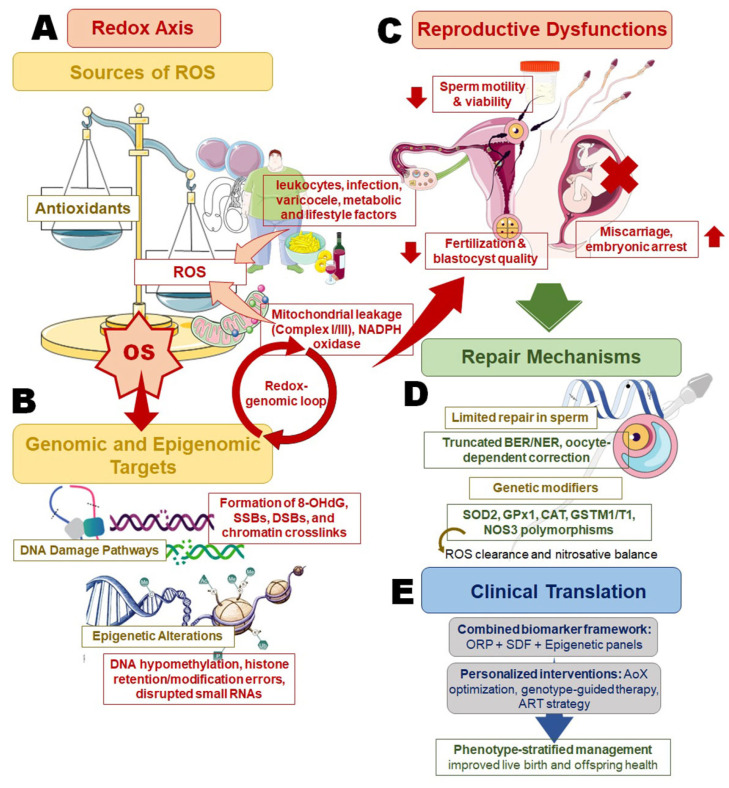
Redox–genomic–epigenomic crosstalk in male infertility and its implication in personalized management. This schematic summarizes the sequential and interlinked mechanisms through which oxidative stress (OS) contributes to male infertility, with each panel highlighting a distinct but interconnected level of regulation. (**A**) The redox axis illustrates major endogenous and exogenous sources of reactive oxygen species (ROS), including mitochondrial electron leakage, NADPH oxidase activity, leukocytic infiltration, varicocele, metabolic dysfunction, and lifestyle-related factors, tipping the balance toward OS when antioxidant capacity is exceeded. (**B**) Genomic and epigenomic targets of OS are shown, emphasizing oxidative DNA lesions (8-OHdG), single- and double-strand breaks (SSBs, DSBs), chromatin crosslinks, and epigenetic dysregulation (DNA hypomethylation, histone retention/modification errors, and altered small RNA profiles), which together establish a self-perpetuating redox–genomic loop. (**C**) The downstream reproductive consequences include impaired sperm motility and viability, reduced fertilization and blastocyst development, and increased risk of miscarriage or embryonic arrest. (**D**) Repair mechanisms are depicted to highlight the biological constraints of sperm DNA repair, reliance on truncated base-excision repair pathways and oocyte-mediated correction, and modulation by genetic polymorphisms affecting redox balance (e.g., SOD2, GPx1, CAT, GSTM1/T1, NOS3). (**E**) The clinical translation panel integrates these mechanisms into a precision-andrology framework, where combined biomarker assessment (oxidation–reduction potential, sperm DNA fragmentation, and epigenetic profiling) guides antioxidant optimization, genotype-informed therapy, and phenotype-stratified management to improve reproductive outcomes and offspring health.

ROS generation during spermatogenesis represents both a physiological necessity and a potential pathological threat (Figure 1). Within the seminiferous epithelium, germ cells undergo constant metabolic and structural transformations, rendering them naturally susceptible to fluctuations in OS [17]. The mitochondria are the principal endogenous source. In developing spermatogonia and spermatocytes, oxidative phosphorylation within the inner mitochondrial membrane releases superoxide anions primarily through complexes I and III of the electron transport chain [19]. Under normal conditions, these species are rapidly converted to hydrogen peroxide and water by antioxidant enzymes. However, any imbalance in mitochondrial efficiency, triggered by hypoxia, nutrient stress, or inflammatory cytokines, magnifies electron leakage, escalating ROS accumulation [20]. As spermatids undergo elongation and mitochondrial condensation around the midpiece, ROS production becomes a tightly regulated signaling event that modulates chromatin remodeling, protamine incorporation, and disulfide bond formation within the sperm tail [21]. Paradoxically, the mitochondria that provide energy for sperm motility also constitute their primary vulnerability. Due to their restricted cytoplasmic volume and deficient repair mechanisms, mature spermatozoa are ill-equipped to withstand prolonged OS [22].

Beyond mitochondrial metabolism, several extrinsic and somatic factors amplify ROS exposure. Leukocytic infiltration in semen, particularly activated neutrophils and macrophages, can release large bursts of superoxide and hypochlorous acid [23]. Testicular and epididymal epithelial cells may also generate ROS through Nicotinamide Adenine Dinucleotide Phosphate (NADPH) oxidases (NOX family), xanthine oxidase, or cytochrome P450 systems [24]. Lifestyle-related triggers, tobacco smoke, alcohol, obesity-related inflammation, and environmental pollutants such as phthalates or heavy metals, introduce exogenous oxidants that further burden the delicate redox equilibrium [25]. Even physiological events, such as capacitation, transiently enhance ROS production. However, when unchecked, these same processes evolve into pathological OS [5].

Seminal plasma composition plays a decisive buffering role. Albumin, ascorbate, and urate scavenge free radicals, but their efficacy declines with advancing age or systemic metabolic disorders [26]. The unique microenvironment of the epididymis, which normally supports redox-dependent maturation, may also become a site of oxidative injury under infection or heat stress [27]. Thus, both mitochondrial and external sources of ROS generate a complex scenario of OS throughout spermatogenesis, requiring a finely tuned antioxidant defense system to maintain sperm functionality and genomic stability.

### 3.2. Environmental and Metabolic ROS Amplifiers

Modern lifestyles overlay spermatogenic physiology with continuous oxidative burden. Environmental exposures, heat, radiation, xenobiotics, and endocrine-disrupting chemicals, act as ROS amplifiers, turning subtle redox imbalances into chronic oxidative states [28]. Elevated scrotal temperature from tight clothing, laptop use, or prolonged sitting disrupts mitochondrial respiration, producing superoxide bursts that overwhelm testicular antioxidants [29]. Occupational exposure to pesticides, solvents, or heavy metals further enhances NADPH oxidase activity in Sertoli and Leydig cells [30]. Cigarette smoke is perhaps the most pervasive amplifier. It introduces cadmium, nicotine, and polyaromatic hydrocarbons that generate both direct and indirect ROS. Sperms from smokers exhibit higher levels of lipid peroxidation, 8-hydroxy-2′-deoxyguanosine (8-OHdG), and fragmented DNA, even after cessation, suggesting persistent mitochondrial and epigenetic injury [31]. Alcohol exerts dual oxidative mechanisms, acetaldehyde accumulation and depletion of hepatic and testicular glutathione, both culminating in increased SDF [32].

Obesity and diabetes add a powerful metabolic dimension. Adipose tissue is an active endocrine organ, secreting Tumor Necrosis Factor-alpha (TNF-α), interleukin-6 (IL-6), and leptin, which stimulate ROS production via NOX enzymes [33]. Hyperglycemia induces advanced glycation end products (AGEs) that, upon binding to RAGE receptors in testicular tissue, initiate chronic oxidative-inflammatory loops. These metabolic perturbations suppress antioxidant gene transcription, reduce sperm protamination, and directly elevate DNA fragmentation rates [33,34]. Air pollution, particularly fine particulate matter and ozone, introduces oxidative radicals capable of translocating into the testicular microcirculation [35]. Studies in urban populations consistently link high environmental pollution indices with elevated seminal ROS and decreased sperm motility [36]. Even electromagnetic radiation from mobile phones has been implicated in mitochondrial dysfunction and ROS elevation, though mechanistic consensus remains debated [37].

The common denominator across these diverse amplifiers is mitochondrial stress coupled with depletion of redox buffering systems. Importantly, genetic polymorphisms in antioxidant or detoxification genes, such as glutathione S-transferase Mu 1 (GSTM1), glutathione S-transferase Theta 1 (GSTT1), and superoxide dismutase 2 (SOD2), modulate individual vulnerability, offering a genetic substrate for personalized susceptibility [38]. The convergence of environmental and metabolic ROS amplifiers defines a global trend in male infertility with OS as a lifestyle-linked epidemic, bridging environmental toxicology, metabolism, and reproductive genomics.

### 3.3. Antioxidant Defense Networks

The testis is among the most metabolically active organs, and its survival depends on a multilayered antioxidant system operating in both somatic and germ cells [39]. This defense network comprises enzymatic and non-enzymatic components distributed across distinct compartments of the seminiferous epithelium and seminal plasma [40]. At its enzymatic core lie SOD, catalase, and glutathione peroxidase (GPx). SOD catalyzes the dismutation of superoxide to hydrogen peroxide, which is subsequently neutralized by catalase or GPx into water. Each enzyme occupies specialized location with SOD1 in cytoplasm, SOD2 within mitochondria, and SOD3 in extracellular spaces, thereby ensuring spatial containment of ROS cascades [28]. Glutathione (GSH) operates as a ubiquitous redox buffer, maintaining thiol groups in a reduced state critical for chromatin protamination and sperm membrane fluidity. The GSH/GSSG ratio is dynamically sustained through the action of glutathione reductase, powered by NADPH derived from the pentose phosphate pathway [41]. Peroxiredoxins (PRDXs) and thioredoxin systems provide additional peroxide detoxification and redox signaling control, particularly within the post-meiotic germ cells where DNA packaging is underway. These proteins not only scavenge peroxides but also act as redox sensors, influencing transcriptional regulators that determine germ cell survival or apoptosis [14].

Non-enzymatic antioxidants complement this enzymatic shield. Vitamins C and E act synergistically, ascorbate regenerating the reduced form of α-tocopherol to protect lipid membranes from peroxidation [42]. Trace elements like selenium and zinc serve as structural cofactors for antioxidant enzymes and sperm chromatin stability, while other micronutrients, including phosphate, modulate cellular redox balance by influencing mitochondrial function, energy metabolism, and oxidative signaling pathways [43]. Beyond systemic redox regulation, the trace element profile of seminal plasma reflects gland-specific biochemical contributions, particularly from the prostate and seminal vesicles, indicating that oxidative imbalance may arise from disproportionate glandular activity and could be addressed through targeted, organ-informed micronutrient or antioxidant modulation [44]. In parallel, epididymal fluid and seminal plasma further reinforce the antioxidant milieu through molecules such as carnitine, taurine, uric acid, and albumin [45]. However, this equilibrium is sensitive to internal and external influences as age, hormonal imbalance, excess adiposity, and xenobiotic exposure can all compromise antioxidant defenses by downregulating gene expression or depleting vital reserves [4,25]. Polymorphisms in genes encoding SOD2, GPx4, or PRDX6 have been associated with altered seminal oxidative profiles, underscoring genetic vulnerability [46]. Importantly, an excess of antioxidants may paradoxically induce a ‘reductive stress’, disturbing physiological ROS signaling required for sperm maturation [47]. Accordingly, antioxidant defense represents not a static shield but a dynamic redox balance that maintains the delicate balance between physiological and pathological levels of ROS throughout spermatogenesis.

### 3.4. Molecular Targets of Oxidative Damage

ROS are spontaneous in their reactivity, and within spermatozoa, virtually every macromolecule is a potential target. Lipid peroxidation is the most visible manifestation, driven by hydroxyl radicals attacking polyunsaturated fatty acids in sperm membranes. The resultant malondialdehyde and 4-hydroxynonenal adducts alter membrane fluidity, compromise ion channel function, and impair fusion with the oocyte [48]. Protein oxidation follows closely, leading to carbonylation, cross-linking, and loss of enzymatic activity [21]. Axonemal proteins, especially dynein adenosine triphosphatase (ATPase) and tubulin, are highly susceptible, resulting in diminished motility and defective flagellar movement patterns [49]. However, it is the damage to nucleic acids that exhibits the most profound reproductive consequence. OS induces base modifications such as 8-OHdG, single- and double-strand breaks, and chromatin cross-links [6]. Because histone-protamine transition during spermiogenesis is incomplete in a subset of sperm, histone-retained domains remain particularly exposed to ROS attack [50]. These regions are often enriched in developmental and imprinting genes, suggesting that oxidative lesions could transmit epigenetic instability to the embryo [7].

The mitochondrial genome represents another vulnerable target. Devoid of protective histones and possessing only limited repair capacity, mtDNA accumulates mutations at a rate several times greater than that of nuclear DNA [51]. Such mutations can disrupt ATP synthesis and enhance ROS generation further, a vicious self-propagating cycle [52]. Additionally, oxidation of phospholipids in the mitochondrial sheath affects membrane potential, compromising sperm motility and viability [53]. Epigenetic machinery also reacts sensitively to oxidative environments. Oxidation of methyl-cytosine or damage to ten-eleven translocation (TET) enzymes can distort methylation landscapes, while redox imbalance alters histone acetylation through modulation of sirtuins and histone acetyltransferases (HATs) [54]. These cumulative modifications underscore the close association of oxidative and epigenetic injury, hinting at a shared biochemical origin that may underlie transgenerational effects. The testis, thus, stands as a paradoxical environment, where ROS act as indispensable messengers for maturation, yet their excess unleashes a cascade of molecular injuries capable of disabling reproductive potential.

### 3.5. Repair Limitations in Sperm

Despite continuous oxidative damage, the ability of sperms to repair DNA and protein damage is strikingly constrained. During spermatogenesis, meiotic and post-meiotic germ cells possess standard repair pathways, base excision repair (BER), nucleotide excision repair (NER), and homologous recombination (HR), that function efficiently in early stages [55]. However, as chromatin condensation intensifies and cytoplasm is extruded, the molecular machinery for DNA repair is progressively dismantled [56]. Enzymes such as 8-oxoguanine DNA glycosylase 1 (OGG1) or poly (ADP-ribose) polymerase 1 (PARP1) may persist in truncated forms, but the absence of downstream effectors like XRCC1 or DNA polymerase β renders these pathways incomplete [57]. Mature spermatozoa, therefore, enter ejaculation with limited capacity to resolve oxidative lesions. They rely instead on oocyte-mediated repair post-fertilization. Oocytes can excise certain oxidative bases and rejoin single-strand breaks in sperm DNA, yet when paternal damage exceeds a threshold, embryonic checkpoints trigger arrest or apoptosis. This dependency places male fertility not solely on the sperm but also in the reparative competence of the female gamete [51,57]. Protein and lipid repair systems show similar constraints. Methionine sulfoxide reductase (Msr) activity is minimal in mature sperm, allowing oxidized amino acids to accumulate [58]. Phospholipid remodeling enzymes are also scarce, signifying once peroxidized, membrane components cannot be restored. The lack of transcriptional and translational capacity seals this vulnerability, what is lost cannot be replenished.

Clinical implications are far-reaching. Elevated sperm DNA fragmentation index (DFI) or high 8-OHdG levels often correlate with recurrent pregnancy loss and poor ART outcomes, reflecting unrepaired oxidative insults [54]. Moreover, men with varicocele, diabetes, or obesity show reduced expression of repair genes even at the spermatogonial level [59,60,61], indicating systemic metabolic suppression of genomic maintenance. Thus, repair limitations are not mere technical footnotes but a defining characteristic of sperm biology. They convert transient oxidative fluctuations into lasting genomic scars, bridging the gap between environmental exposures and heritable reproductive failure. Understanding these constraints reinforces the necessity for redox-targeted diagnostics and interventions that protect sperm DNA before damage becomes irreversible, a central theme guiding the evolution of personalized andrological medicine.

## 4. Mechanistic Routes to SDF

**Figure 2:**
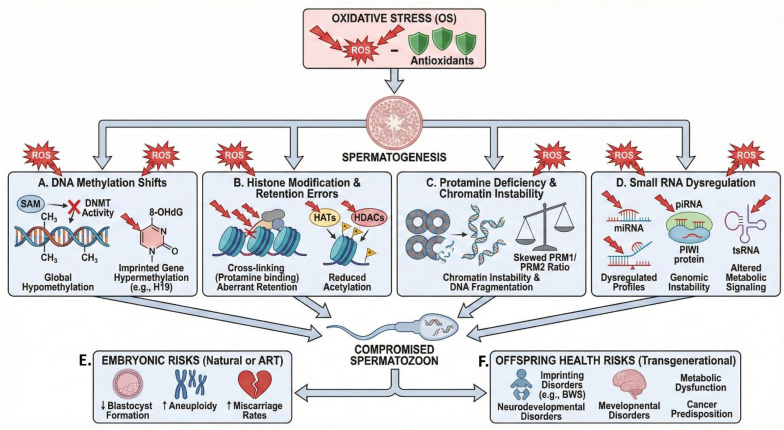
Mechanisms and consequences of oxidative stress-induced epigenetic dysregulation in male reproduction. Excessive reactive oxygen species (ROS) induce oxidative stress (OS), which disrupts spermatogenesis through four interconnected epigenetic pathways. (**A**) OS alters DNA methylation by oxidizing S-adenosylmethionine (SAM) and impairing DNA methyltransferase (DNMT) and TET enzyme activity, resulting in global hypomethylation together with site-specific hypermethylation at imprinted loci. (**B**) ROS promote histone carbonylation and protein cross-linking and inhibit histone acetyltransferases (HATs), leading to aberrant histone retention and reduced histone acetylation required for proper paternal genome activation. (**C**) OS disrupts protamine expression and processing, altering the PRM1/PRM2 ratio and causing chromatin instability and increased DNA fragmentation. (**D**) Sperm small non-coding RNAs (miRNA, piRNA, and tsRNA) function as OS sensors; their dysregulation under oxidative conditions compromises genomic integrity and perturbs metabolic signaling. Collectively, these epigenetic abnormalities generate functionally compromised spermatozoa that, following natural or assisted fertilization, increase the risk of (**E**) early embryonic developmental failure and (**F**) long-term, potentially transgenerational, health disorders in the offspring.

SDF represents a key molecular signature connecting OS to reduced male fertility. It reflects the occurrence of single- and double-strand breaks or base alterations within sperm chromatin, undermining genomic integrity, fertilization capacity, and embryonic development [62]. The etiology of SDF is multifactorial and includes aberrant chromatin remodeling during spermiogenesis, oxidative DNA damage driven by ROS, and the exacerbating effects of environmental and metabolic stressors [55] (Figure 2). Given the limited repair capacity of mature spermatozoa, these cumulative insults contribute to persistent genomic and epigenomic disruptions that not only impair fertility but may also exert transgenerational effects on offspring health [57].

### 4.1. Chromatin Remodeling Defects

Spermatogenesis is a process marked by extensive chromatin remodeling, among the most significant nuclear transformations in living systems. During spermiogenesis, histones are sequentially replaced by transition proteins and then by protamines, condensing the paternal genome into a toroidal configuration nearly tenfold denser than somatic chromatin [50]. This extreme compaction not only enables hydrodynamic sperm shape but also shields the paternal DNA from oxidative and enzymatic insults. However, when this remodeling falters, the vulnerability of the sperm genome escalates sharply [63]. The replacement process is highly coordinated by post-translational modifications of histones such as acetylation, ubiquitination, and phosphorylation. These marks relax chromatin and recruit transition proteins (TNP1, TNP2), which transiently occupy the nucleosome before protamines (PRM1, PRM2) take command [64]. Incomplete or delayed protamination leaves extended DNA regions loosely packed, accessible to endonucleases and ROS [21]. Histone retention in 5–15% of sperm DNA is considered physiological, as these domains carry developmental gene promoters. However, excessive retention, often exceeding 20–30%, correlates with elevated DNA fragmentation indices [65]. Genetic or epigenetic disruptions in the genes encoding transition proteins or protamines directly affect this balance. PRM2 deficiency, for instance, destabilizes disulfide crosslinking, creating a redox-sensitive pocket around the DNA helix. Similarly, anomalies in chromatin remodelers such as Bromodomain Testis-specific protein (BRDT), Chromodomain Helicase DNA-binding protein 5 (CHD5), or histone chaperone Nucleosome Assembly Protein 1-like 4 (NAP1L4) impede the eviction of histones, leading to uneven compaction and defective nuclear shaping. The outcome is structurally fragile DNA susceptible to breaks during epididymal transit or ejaculation [66].

Oxidative conditions amplify these defects. ROS oxidize cysteine residues within protamines, impairing proper disulfide bridge formation [67]. This biochemical weakening magnifies mechanical shearing during sperm movement, producing strand breaks even in the absence of exogenous DNA-damaging agents [57]. Moreover, protamine-deficient sperm display higher levels of residual cytoplasm, which houses oxidoreductases like NADPH oxidase, further fueling localized ROS production [68]. Thus, chromatin remodeling defects lie at the core of structural fragility in sperm DNA. They bridge molecular genetics with oxidative biology, showing that DNA fragmentation is rarely random but the predictable outcome of flawed nuclear condensation under oxidative duress. Recognizing these defects has clinical value, as protamine ratios and histone retention patterns are now being explored as biomarkers of sperm nuclear maturity and fertilization competence.

Beyond nuclear compaction defects, OS also exerts its effects through redox-induced modifications of sperm proteins that operate during epididymal transit and ejaculation, phases characterized by heightened oxidative vulnerability. In spermatozoa retaining excess residual cytoplasm, redox-sensitive metabolic and structural proteins are particularly prone to oxidative modification, impairing energy homeostasis, cytoskeletal stability, and protein-DNA interactions [58]. While such redox modifications may initially represent adaptive responses to oxidative pressure, their persistence under chronic OS can indirectly exacerbate sperm DNA fragmentation by compromising chromatin support mechanisms and limiting effective protection during mechanical stress. From a personalized management perspective, profiling redox-induced protein states may therefore help distinguish adaptive redox responses from pathological oxidative injury, providing mechanistic context to elevated SDF beyond chromatin remodeling defects alone.

### 4.2. Oxidative DNA Lesions and Breaks

Once chromatin architecture is compromised, sperm DNA becomes a prime target for oxidative assault. ROS attack guanine bases preferentially, forming oxidized adducts such as 8-OHdG [69]. This lesion mis-pairs with adenine during replication, introducing G-to-T transversions in the zygote if unrepaired by the oocyte. Hydrogen peroxide and hydroxyl radicals can also induce single-strand breaks (SSBs) through direct sugar oxidation, and clustered oxidative events may evolve into double-strand breaks (DSBs) [70]. Unlike somatic cells, spermatozoa cannot mount a full DNA-damage response. The absence of nucleosomes and canonical repair enzymes means that oxidative lesions persist unrepaired until fertilization [71]. Consequently, the oocyte takes on the burden of excising 8-OHdG and rejoining nicks; however, oocyte repair efficiency declines with maternal age, magnifying the reproductive risk of paternal oxidative damage [72].

Mitochondrial dysfunction contributes another layer of complexity. Electron leakage from the mitochondrial electron transport chain produces superoxide, which diffuses toward the nucleus through the annulus and midpiece [73]. Mitochondrial ROS not only inflict direct oxidative lesions but also trigger apoptotic-like pathways in sperm, including activation of caspase-dependent endonucleases such as endonuclease G (EndoG) and apoptosis-inducing factor (AIF). These enzymes translocate to the nucleus, cleaving DNA into 180–200 bp fragments reminiscent of apoptosis in somatic cells [74]. OS is also synergized by transition metals. Elevated iron or copper levels in semen catalyze the Fenton reaction, generating hydroxyl radicals that cause rapid strand scission. This explains why varicocele, infection, or hemato-testicular barrier disruption, all associated with metal accumulation, show higher SDF [75,76].

An emerging dimension involves redox-mediated epigenetic drift. Oxidative base modifications can inhibit DNA methyltransferase (DNMT) binding, leading to local hypomethylation and altered chromatin accessibility [77]. Such regions are more prone to cleavage, reinforcing the oxidative–epigenetic nexus in SDF. In this way, oxidative DNA injury should be seen as a gradual process rather than an isolated event, progressing from small oxidative modifications to major strand breaks that influence genome stability and heritable mutation risk.

### 4.3. Clinical Consequences of SDF

The clinical footprint of SDF extends well beyond laboratory indices. Elevated SDF signifies compromised paternal genomic integrity that directly affects fertilization, embryo development, implantation, and pregnancy outcomes [78]. Men with high SDF may present with normal semen parameters, revealing the inadequacy of conventional semen analysis in assessing true reproductive potential. Natural conception rates decline progressively as SDF exceeds critical thresholds (typically > 25–30%), with increased risk of early miscarriage [79]. In assisted reproduction, intracytoplasmic sperm injection (ICSI) bypasses motility and morphology barriers but not genetic quality [80]. High SDF correlates with lower blastocyst formation rates, increased aneuploidy, and reduced live-birth outcomes even after ICSI [81]. The capacity of the oocyte to repair sperm DNA breaks determines success; when this capacity wanes, as in advanced maternal age, the risk of embryonic developmental arrest or implantation failure surges [82].

Beyond immediate fertility outcomes, SDF has potential transgenerational implications. Oxidatively damaged sperm can introduce base mutations and epimutations that escape embryonic reprogramming, predisposing offspring to neurodevelopmental or metabolic disorders [83]. Animal studies show paternal oxidative damage influencing offspring glucose metabolism, brain function, and telomere length, echoing the human concern for intergenerational health effects [84,85].

Clinically, SDF testing is increasingly adopted to stratify male infertility phenotypes and tailor interventions. Patients with varicocele or infection show SDF improvement after surgical or medical correction, whereas those with idiopathic OS may benefit from targeted antioxidant regimens [86]. However, therapeutic efficacy depends on redox profiling: indiscriminate antioxidant use risks reductive imbalance and impaired sperm signaling [87]. SDF assessment, via Terminal deoxynucleotidyl transferase dUTP nick end labeling (TUNEL), Comet, or sperm chromatin structure assay, thus provides a bridge between laboratory diagnostics and personalized therapy [88]. Integrating SDF metrics with OS markers and protamine ratios offers a multidimensional evaluation of sperm health [89]. In the emerging landscape of precision andrology, SDF is no longer a secondary biomarker but a sentinel indicator linking redox biology to genomic and epigenetic integrity. Its recognition reshapes how clinicians interpret male infertility, not merely as a failure of sperm count or motility, but as a reflection of systemic oxidative dysregulation reverberating through the paternal genome.

## 5. Epigenetic Dysregulation Under OS

### 5.1. Oxidative Shifts in DNA Methylation

Oxidative stress profoundly disrupts DNA methylation patterns in spermatozoa, creating heritable epigenetic alterations that compromise male fertility and offspring health (Figure 1). ROS directly oxidize the methyl group donor S-adenosylmethionine (SAM), reducing its bioavailability and subsequently impairing DNA (cytosine-5)-methyltransferase (DNMT) activity [90]. This ROS-mediated depletion of SAM creates a cascade effect whereby global DNA hypomethylation occurs alongside region-specific hypermethylation at CpG islands, particularly affecting imprinted genes critical for embryonic development [90,91].

The oxidative modification of cytosine bases represents another critical mechanism linking OS to methylation dysregulation. ROS-induced formation of 8-OHdG and 5-hydroxymethylcytosine (5-hmC) interferes with normal methylation maintenance during spermatogenesis [92]. Studies demonstrate that infertile men with elevated seminal ROS levels exhibit significantly altered methylation patterns at paternally imprinted loci, including H19/Insulin-like Growth Factor 2 (H19/IGF2), Mesoderm Specific Transcript (MEST), and Small Nuclear Ribonucleoprotein Polypeptide N (SNRPN), compared to normozoospermic controls [92,93]. These aberrant methylation signatures correlate strongly with high sperm DFI values, suggesting a mechanistic link between oxidative damage and epigenetic instability [94].

Furthermore, OS compromises the activity of TET enzymes, which catalyze the oxidation of 5-mC to 5-hmC in the demethylation pathway [95]. Clinical investigations reveal that antioxidant interventions, including vitamins C and E, coenzyme Q10, and N-acetylcysteine, can partially restore normal methylation patterns in patients with abnormal sperm parameters [96]. Notably, varicocele-associated OS has been linked to hypomethylation of the Methylenetetrahydrofolate reductase (MTHFR) gene promoter, creating a vicious cycle that further impairs folate metabolism and methylation capacity [97,98]. These findings underscore the critical importance of assessing methylation status alongside conventional semen parameters in the personalized management of OS-mediated male infertility.

### 5.2. Histone Modification and Retention Errors

During spermiogenesis, approximately 85–95% of histones are replaced by protamines to achieve the extreme chromatin condensation characteristic of mature spermatozoa; however, the retained histones play crucial regulatory roles in early embryonic gene expression [65,99]. Oxidative stress severely disrupts this histone-to-protamine transition, resulting in aberrant histone retention patterns that compromise both chromatin packaging and epigenetic information transfer [15]. Elevated ROS levels induce excessive histone carbonylation and oxidative cross-linking, impeding the proper displacement of histones by PRM1 and PRM2 during spermatid elongation [68,100].

Post-translational histone modifications (PTMs), including acetylation, methylation, phosphorylation, and ubiquitination, serve as critical epigenetic marks that regulate chromatin accessibility and gene expression [101]. Oxidative stress-mediated oxidation of HATs and histone deacetylases (HDACs) alters the acetylation landscape, with infertile men exhibiting significantly reduced H3K9ac and H3K14ac levels compared to fertile controls [102,103]. These acetylation deficits correlate with impaired chromatin relaxation during fertilization and compromised paternal genome activation in early embryos [104].

### 5.3. Protamine Deficiency and Chromatin Instability

Protamines represent the cornerstone of sperm chromatin architecture, replacing histones to achieve DNA packaging density approximately 6-fold greater than somatic cells, thereby protecting paternal genetic material during transit through the male and female reproductive tracts [99,105]. Oxidative stress fundamentally compromises protamine expression, processing, and DNA binding, creating structural instabilities that predispose to DNA fragmentation and fertility failure [106]. The PRM1/PRM2 ratio, optimally maintained at approximately 1:1 in fertile men, becomes significantly skewed under OS conditions, with many infertile patients exhibiting ratios exceeding 1.5:1 or falling below 0.8:1 [107,108].

Advanced techniques including chromatinomics and proteomic profiling reveal that OS-mediated protamine insufficiency affects specific genomic regions non-randomly, with preferential vulnerability at GC-rich sequences and repeat elements [44,45]. The aniline blue (AB) and chromomycin A3 (CMA3) tests, which detect protamine-deficient chromatin, show significantly elevated staining in men with varicocele, obesity, and environmental toxin exposure; all conditions associated with heightened OS [109,110,111]. Clinical interventions targeting OS reduction through antioxidant supplementation have demonstrated improvements in protamine content, with documented normalization of PRM1/PRM2 ratio following 3–6 months of treatment with combined vitamin E, selenium, and carnitine regimens [112,113].

### 5.4. Small RNAs as OS Sensors

The sperm epigenome encompasses a diverse repertoire of small non-coding RNAs, including microRNAs (miRNAs), PIWI-interacting RNAs (piRNAs), and transfer RNA-derived small RNAs (tsRNAs), which function as both OS sensors and mediators of epigenetic information to offspring [114,115]. These regulatory molecules, comprising the majority of sperm RNA content, exhibit remarkable sensitivity to oxidative perturbations, with their expression profiles serving as molecular signatures of paternal environmental exposures [115,116]. MicroRNAs, particularly those involved in DNA methylation regulation (miR-29 family) and OS response (miR-34c, miR-122), show significant dysregulation in men with elevated seminal ROS, creating a mechanistic link between OS and epigenetic transgenerational effects [117].

piRNAs maintain genomic integrity by silencing transposable elements [118,119]. Oxidative stress disrupts piRNA biogenesis through impairment of the PIWI protein family (PIWIL1-4), resulting in transposon reactivation and genomic instability [120,121].

Transfer RNA-derived small RNAs, emerge as novel OS biomarkers and potential mediators of acquired metabolic and stress phenotypes to F1 offspring [122,123]. Animal models demonstrate that paternal high-fat diet or chronic stress exposure alters sperm tsRNA profiles, subsequently affecting offspring metabolic regulation and stress response pathways [123,124]. Human studies reveal a correlation between seminal tRNA-derived fragments in semen of normozoospermic men and success rates of ARTs, highlighting a link with unexplained infertility [125]. The expression profile of sperm tRNA-derived fragments changes with shorter abstinence period, with lower sperm DNA fragmentation and higher sperm motility, suggesting a direct link with OS [126].

### 5.5. Embryonic and Offspring Risks

The transgenerational consequences of OS-induced sperm epigenetic aberrations extend beyond fertilization failure, creating substantial risks for embryonic development, pregnancy outcomes, and offspring health across multiple generations [15]. Spermatozoa bearing oxidatively damaged DNA and dysregulated epigenetic marks can successfully fertilize oocytes, particularly with ARTs, yet harbor latent defects that manifest during embryogenesis or later offspring development [127,128]. Studies utilizing ICSI demonstrate that elevated paternal SDF indices associate with significantly reduced blastocyst formation rates, increased embryonic aneuploidy, higher miscarriage rates, and lower live birth rates despite successful fertilization [127,129,130,131].

The mechanistic basis for these developmental failures involves multiple interconnected pathways. Aberrant DNA methylation patterns, particularly at imprinted genes (H19, IGF2, SNRPN, KCNQ1OT1), escape the normal post-fertilization epigenetic reprogramming, resulting in imprinting disorders including Beckwith-Wiedemann syndrome and Silver-Russell syndrome in offspring [132]. Meta-analyses indicate a 2–11-fold increased risk of multiple imprinting disorders in children conceived via ARTs [133,134].

Epidemiological evidence increasingly links paternal OS exposure, through obesity, smoking, advanced age, or environmental toxins, with offspring neurodevelopmental disorders, metabolic dysfunction, and cancer predisposition [135,136,137]. Animal models demonstrate that paternal OS exposure induces behavioral abnormalities, altered stress responses, and metabolic dysregulation in F1 and F2 generations through sperm RNA-mediated epigenetic inheritance [123,138]. Human cohort studies reveal associations between paternal cigarette smoking and increased childhood cancer, and advanced paternal age and offspring autism spectrum disorder [137,139]. These findings underscore the critical importance of comprehensive OS assessment and management prior to natural conception or ART, with emerging guidelines recommending SDF testing, antioxidant optimization, and consideration of testicular sperm extraction in severe cases to minimize transgenerational epigenetic risks [55,140,141].

## 6. Measurement: From Bench to Clinic

The clinical application of redox–genomic knowledge in male infertility hinges on the development of reliable, standardized, and clinically meaningful biomarkers. Conventional semen analysis, though valuable for assessing morphology and motility, provides limited insight into the molecular and oxidative aspects of sperm dysfunction [142]. Integrating redox, genomic, and epigenetic profiling provides a deeper mechanistic framework for phenotype-specific diagnosis and management. Evolving from conventional biochemical assays to modern biosensing technologies, current approaches now capture OS, SDF, and epigenetic irregularities as overlapping dimensions of male reproductive health.

### 6.1. Redox Biomarkers and Oxidation Stress Testing

The transition from understanding OS conceptually to measuring it quantitatively has been pivotal in modern andrology. Redox biomarkers bridge the gap between biochemical insight and clinical decision-making, offering tangible indices of oxidative balance within semen (Table 1). Traditionally, OS was inferred indirectly from surrogate parameters such as lipid peroxidation or antioxidant enzyme activities [143]. However, these discrete assays often failed to reflect the dynamic interplay between oxidants and reductants that defines physiological redox homeostasis [18].The emergence of oxidation–reduction potential (ORP) testing has refined this landscape. ORP quantifies the integrated redox state of a sample, representing the balance between all electron donors and acceptors [144]. In seminal plasma, a higher ORP value indicates a net oxidative shift, signaling depletion of antioxidant reserves or overproduction of ROS [145]. Results of the ORP test has been correlated with SDF, motility, and morphology [146]. However, ORP is not a stand-alone metric. Complementary biomarkers enrich interpretation. 8-OHdG reflects oxidative DNA lesions [147], while malondialdehyde and 4-hydroxynonenal (4-HNE) quantify lipid peroxidation [148]. Protein carbonyls, nitrotyrosine, and isoprostanes extend the oxidative footprint to the proteome and metabolome [149]. Enzymatic assays for SOD, glutathione peroxidase GPx, and catalase delineate the defensive arm of the redox equation, whereas total antioxidant capacity integrates them into a functional measure of seminal antioxidative capacity [150].

Standardization remains a major limiting factor in the measurement and clinical translation of redox biomarkers for male infertility. Differences in sampling, centrifugation, or cryostorage can alter redox readings dramatically. Recognizing this, the World Health Organization (2021) has emphasized harmonized pre-analytical protocols and temperature-controlled measurements [151]. Population-specific reference intervals are also necessary, as baseline ORP and antioxidant enzyme levels exhibit ethnic, dietary, and climatic variation [151]. Clinically, redox biomarkers serve multiple purposes: diagnostic (identifying oxidative infertility), prognostic (predicting ART outcomes), and therapeutic (monitoring response to antioxidant or lifestyle interventions). Elevated ORP values often signify clinically significant OS, guiding the use of targeted antioxidant therapy rather than empirical supplementation [145].

In the era of personalized medicine, combining ORP with traditional semen parameters and SDF indices allows stratification into distinct redox–genomic phenotypes, informing individualized therapeutic algorithms. Ultimately, redox biomarker testing has evolved from bench-based experimentation to bedside application. What once demanded sophisticated instrumentation can now be integrated into routine andrology labs, although most current assays still await rigorous validation and standardization before oxidative stress assessment can be considered a reliable, actionable clinical tool [152]. Clinical evidence increasingly indicates that the efficacy of antioxidant therapy in male infertility is contingent upon baseline redox status. Men with objectively elevated OS demonstrate significantly greater improvements in sperm DNA integrity and reproductive outcomes following antioxidant intervention, whereas empiric supplementation in normo-redox individuals may confer limited benefit or induce reductive imbalance [153]. These findings support the use of redox biomarkers as a prerequisite for treatment selection, positioning oxidative profiling as a determinant of therapeutic responsiveness rather than a post hoc explanatory measure.

### 6.2. Assays for SDF

Quantifying SDF has redefined the diagnostic reach of andrology, providing insight into male infertility that conventional semen analysis often overlooks [55]. Multiple assays now exist, differing in principle, sensitivity, and clinical interpretability, yet united by the goal of gauging genomic integrity at the single-cell level (Table 1). The TUNEL assay remains a gold standard in research. It directly labels free 3′-OH termini of DNA breaks using fluorescent dUTP analogs. Flow cytometric TUNEL offers quantitative precision, while fluorescence microscopy enables morphological correlation. Although highly sensitive to both single- and double-strand breaks, TUNEL demands meticulous standardization and skilled operators and expensive equipment, factors that limit its widespread clinical deployment [88]. The Sperm Chromatin Structure Assay (SCSA), developed by Evenson and colleagues, measures the susceptibility of sperm DNA to acid-induced denaturation using acridine orange staining, distinguishing native double-stranded DNA (green fluorescence) from denatured single-stranded regions (red fluorescence) [56]. The derived DFI correlates robustly with fertilization and pregnancy outcomes, with thresholds of 25–30% often predicting subfertility [79,81]. Reproducibility of SCSA and large-scale reference data make it the most validated clinical assay, though it reflects chromatin instability more than direct breaks [88]. The Comet assay, a single-cell gel electrophoresis method, provides visualization of DNA migration resembling a ‘comet tail’ [88]. The tail length and intensity quantify strand breakage, with alkaline and neutral variants distinguishing SSBs and DSBs, respectively. Its sensitivity to oxidative lesions makes it particularly useful in experimental and toxicological studies. However, variability in electrophoresis conditions and scoring algorithms hinders clinical harmonization [55].

Newer technologies have extended analytical scope. Sperm chromatin dispersion test uses controlled denaturation and lysis to visualize DNA halos under bright-field microscopy, offering a cost-effective yet semi-quantitative alternative for clinical labs [154]. Meanwhile, Flow-TUNEL and high-content imaging platforms integrate automation, enabling high-throughput SDF screening. Interpretation of SDF data must account for confounders: abstinence duration, leukocytospermia, infections, and fever can transiently elevate fragmentation levels [155]. Moreover, assay-specific cutoffs differ, emphasizing the need for laboratory-specific validation. Clinical translation is most meaningful when SDF results are combined with OS and protamine status assessments, generating a multidimensional view of sperm health. Therapeutically, SDF assays serve as benchmarks for intervention efficacy, declines in DFI or TUNEL positivity after varicocelectomy, infection control, or antioxidant therapy indicate successful genomic stabilization [156,157,158]. In ART, SDF testing can guide sperm selection techniques such as microfluidic sorting or magnetic-activated cell sorting, improving embryo quality [159]. Therefore, SDF measurement has evolved from a niche research tool into a critical biomarker for personalized fertility management, bridging the mechanistic insights of redox–genomic interplay with the tangible metrics of clinical prognosis.

### 6.3. Epigenetic and Chromatin Assays

Epigenetic and chromatin analyses have redefined how sperm quality is assessed, extending far beyond the classical measures of motility and morphology to capture deeper molecular aspects of fertility potential. Unlike SDF, which assesses physical DNA breaks, these tests interrogate the regulatory blueprint that governs gene expression and developmental competence. Epigenetic modifications, DNA methylation, histone post-translational marks, and small non-coding RNAs, act as heritable molecular memories of environmental and oxidative experiences [160].

DNA methylation profiling forms the cornerstone of sperm epigenetic assessment. Bisulfite conversion followed by sequencing (bisulfite-seq) or array-based methods enables quantification of 5-methylcytosine at single-base resolution [161]. Hypomethylation at imprinted loci such as H19 or MEST and global methylation drift are recurrent features in men with high OS or idiopathic infertility [162,163]. The advent of targeted methylation panels and next-generation sequencing has transformed this analysis from research-intensive to semi-clinical feasibility, although cost and bioinformatics expertise remain limiting factors. Histone retention and modification assays provide complementary insights [163]. Chromatin immunoprecipitation combined with sequencing identifies specific histone modifications such as H3K4me3 or H3K9me2 that regulate transcriptional competence of the paternal genome [164]. In infertile men, aberrant histone retention is often localized to developmental gene promoters, reflecting disturbed chromatin remodeling during spermiogenesis [165]. Simpler proxies, such as protamine 1-to-2 ratios or AB staining, are used in clinical laboratories to approximate chromatin maturity and correlate with SDF. Small RNA profiling, encompassing microRNAs and piRNAs, adds another dimension to the redox–genomic dialog [166]. OS reshapes sperm RNA cargo, affecting pathways related to early embryogenesis and metabolism [167]. Quantitative RT-PCR or small RNA sequencing can detect these perturbations, offering future biomarkers for paternal epigenetic health [168].

From a translational perspective, integrating epigenetic assays into fertility diagnostics demands careful interpretation. Epigenetic landscapes are inherently dynamic and influenced by age, diet, stress, and environmental exposures [160]. Therefore, single measurements may not fully capture biological variability. Nonetheless, consistent alterations across independent cohorts suggest that oxidative imbalance imprints stable epigenetic scars detectable in sperm. The clinical value of these assays lies in their predictive power. Epigenetic alterations correlate with poor ART outcomes, recurrent pregnancy loss, and offspring health risks. By combining methylation or histone signatures with OS and SDF data, clinicians can classify patients into redox-epigenetic phenotypes, paving the way for precision-guided interventions, ranging from antioxidant optimization to epigenetic-safe ART protocols [169]. Thus, epigenetic and chromatin assays represent the frontier of male infertility diagnostics: not merely measuring damage, but decoding the molecular memories left by OS. As analytic costs decline and reference databases expand, their integration into clinical algorithms will transform male reproductive evaluation from descriptive morphology to predictive molecular medicine.

## 7. Clinical Endotypes: Who Is the ‘Redox–Genomic’ Patient?

Male infertility seldom arises from a single cause, rather, it encompasses a spectrum of intersecting molecular abnormalities that extend beyond simple defects in sperm formation. The growing recognition of redox–genomic crosstalk has shifted the diagnostic lens from descriptive semen parameters toward mechanistic endotyping, the identification of biologically coherent patient subgroups defined by dominant molecular derangements. Within this evolving taxonomy, five broad endotypes have been hypothesized: (i) redox-dominant, (ii) redox-driven DNA fragmentation, (iii) chromatin or epigenetic dysfunction with a redox overlay, (iv) epigenetic-dominant, and (v) SDF-isolated endotype. Each represents a distinct intersection of oxidative metabolism, genomic stability, and clinical phenotype, demanding tailored management rather than uniform empiricism.

### 7.1. Redox-Dominant Endotype

This endotype represents men in whom oxidative imbalance is the primary pathogenic driver. Elevated seminal ORP, lipid peroxidation markers, and depleted antioxidant enzymes precede measurable genomic or epigenetic damage. The clinical phenotype often involves lifestyle or metabolic contributors, smoking, obesity, varicocele, or diabetes, with partially preserved sperm morphology but poor fertilization efficiency. Timely antioxidant or lifestyle correction may help restore function, confirming redox sensitivity as the root dysfunction. These patients illustrate the earliest, reversible phase of redox–genomic perturbation, where genomic damage remains secondary to oxidative burden.

### 7.2. Redox-Driven DNA Fragmentation

Here, OS has crossed the biochemical threshold into structural injury. Elevated 8-OHdG and high SDF index (>30%) indicate direct ROS assault on sperm DNA, with partial failure of antioxidant defenses. Conventional semen parameters are usually abnormal, and fertilization and implantation rates are reduced. This phenotype benefits most from integrated redox and genomic monitoring, using OS and SDF tandem testing, to guide antioxidant or varicocelectomy interventions. Without timely correction, sustained fragmentation promotes chromatin instability and epigenetic divergence, merging oxidative damage with genomic dysfunction.

### 7.3. Chromatin/Epigenetic Dysfunction with Redox Overlay

In this endotype, defective protamination, histone retention, or aberrant methylation patterns pre-exist, rendering chromatin structurally vulnerable. Oxidative stress acts as an accelerant rather than the origin, aggravating SDF and transcriptional instability. Clinically, these men exhibit inconsistent fertility outcomes despite normal or slightly elevated ROS levels, reflecting chromatin fragility that amplifies minor oxidative stimuli. Diagnostic emphasis should include protamine ratio assessment and methylation profiling alongside redox indices. Personalized interventions target both chromatin maturation and redox optimization, highlighting how oxidative signals intersect with inherited or developmental epigenetic weaknesses.

### 7.4. Epigenetic-Dominant Endotype

This phenotype embodies patients whose infertility stems primarily from aberrant sperm epigenetic programming rather than overt oxidative injury. Global DNA hypomethylation, altered imprinting loci, or disturbed histone marks predominate, often linked to advanced paternal age, environmental exposures, or chronic subclinical inflammation. ROS act subtly, modifying methylation enzymes or chromatin remodeling factors, without causing high SDF. These men frequently produce morphologically normal sperm yet contribute to recurrent implantation failure or early miscarriage. Management requires cautious use of antioxidants, metabolic optimization, and consideration of ‘epigenetic-safe’ ART protocols emphasizing minimal gamete manipulation.

### 7.5. SDF-Isolated Endotype

The SDF-isolated category describes individuals with high SDF in the absence of measurable oxidative or epigenetic abnormalities. Causes may include apoptotic dysregulation during spermiogenesis, or cryptic infections producing nuclease activity. Redox biomarkers remain within normal limits, distinguishing this endotype from OS-mediated infertility. Clinically, prognosis depends on the degree of fragmentation and oocyte repair capacity. These cases underscore the heterogeneity of male infertility, reminding clinicians that not all genomic damage is oxidative in origin and advocating multi-axis assessment for precision diagnosis.

Emerging evidence indicates that gut dysbiosis represents an upstream modifier of redox imbalance and sperm epigenetic instability. Dysbiosis-associated endotoxemia and altered microbial metabolites can amplify systemic OS and perturb one-carbon metabolism, thereby influencing sperm DNA integrity and epigenetic programming [170]. These findings support selective consideration of microbiota-targeted strategies as adjuncts within personalized male infertility management, particularly in redox- and epigenetically vulnerable phenotypes.

## 8. Genetic Modifiers of Redox Damage and Repair

### 8.1. Antioxidant Enzyme Polymorphisms

The antioxidant defense machinery of the testis is an intricately coordinated system that maintains the redox balance essential for spermatogenesis. Its efficiency, however, is not uniform across individuals [171]. Polymorphisms in genes encoding antioxidant enzymes can influence catalytic efficiency, predisposing certain men to OS-related sperm dysfunction [172]. Such variants constitute early genetic markers within the redox–genomic framework of male infertility.

Superoxide dismutases represent the first enzymatic barrier against ROS. Among them, SOD2, located in the mitochondrial matrix, catalyzes the conversion of superoxide radicals to hydrogen peroxide [173]. A single nucleotide polymorphism at codon 16 (Val16Ala, rs4880) changes the mitochondrial targeting sequence, altering protein import and activity [174]. The Ala variant, though efficient in transport, displays reduced catalytic capacity, leading to higher oxidative burden within the mitochondria. Numerous studies link the Ala/Ala genotype with elevated SDF, lipid peroxidation, and decreased motility, particularly in men with varicocele or idiopathic infertility [175]. Glutathione peroxidases, especially GPx1 and GPx4, neutralize hydrogen peroxide and lipid hydroperoxides, safeguarding sperm membrane integrity [176]. GPx4 also functions as a structural protein within the mitochondrial sheath. Polymorphisms such as GPx1 Pro198Leu (rs1050450) reduce enzyme activity, compromising sperm membrane fluidity and motility [177]. Similarly, variants in GPx4 promoter regions affect expression levels, correlating with reduced seminal antioxidant capacity [178]. Interestingly, selenium availability modulates the phenotypic impact of these variants, suggesting nutritional-genetic interactions that may determine therapeutic responsiveness to selenium supplementation [179]. Catalase gene polymorphisms also influence redox homeostasis [180]. The –262C>T promoter variant (rs1001179) modifies transcriptional activity, with the T allele linked to lower enzyme expression and increased hydrogen peroxide accumulation in seminal plasma [177]. Men harboring this allele often exhibit higher oxidative SDF and poorer fertilization rates in assisted reproduction. Another pivotal contributor is glutathione S-transferase (GST) family polymorphisms. Deletions in GSTM1 or GSTT1 genes abolish enzyme activity, impairing conjugation of electrophilic intermediates with glutathione [181]. These null genotypes amplify oxidative injury and DNA fragmentation, particularly under environmental exposures such as smoking or air pollution. The cumulative presence of multiple unfavorable alleles across SOD2, GPx1, and GSTM1 constitutes a genetic ‘oxidative haplotype’ that markedly elevates infertility risk [46].

From a translational viewpoint, recognizing antioxidant enzyme polymorphisms transforms management from empirical supplementation to genotype-guided antioxidant therapy. Individuals with SOD2 Ala or GPx1 Leu variants may require tailored antioxidant regimens emphasizing mitochondrial stabilizers (e.g., CoQ10, L-carnitine), whereas those with GSTM1 null genotypes could benefit from thiol-enhancing compounds such as N-acetylcysteine. Future clinical algorithms integrating ORP measurements with genotyping promise to define precise redox–genomic phenotypes. Thus, antioxidant enzyme polymorphisms illustrate how genomic variability reshapes the oxidative landscape of spermatogenesis. They bridge molecular genetics with metabolic phenotype, explaining why identical environmental exposures yield divergent reproductive outcomes. The next challenge lies in incorporating these variants into predictive models that guide personalized interventions in oxidative male infertility.

### 8.2. DNA Repair Gene Variants

Individual susceptibility to oxidative stress-induced SDF exhibits substantial inter-patient variability, largely attributable to single nucleotide polymorphisms (SNPs) in genes encoding DNA repair enzymes and antioxidant defense systems [174]. The 8-oxoguanine DNA glycosylase 1 (OGG1) Ser326Cys polymorphism (rs1052133) represents one of the most extensively studied variants, with the Cys/Cys genotype demonstrating significantly reduced enzymatic activity in excising 8-OHdG lesions compared to Ser/Ser carriers [182]. A recent study on 118 infertile men revealed that OGG1 326Cys allele carriers exhibit significantly elevated SDF and OS indices [182].

The X-ray repair cross-complementing group 1 (XRCC1) gene, encoding a scaffold protein essential for BER pathway coordination, harbors three clinically relevant polymorphisms: GG, GA, and AA. Men carrying the XRCC1 GA and GA + AA variant alleles harbor a higher risk of idiopathic azoospermia [183]. Gene-gene interaction studies reveal synergistic effects, where men carrying multiple risk alleles exhibit increased idiopathic infertility risk compared to protective genotype combinations [184]. Notably, antioxidant response gene variants, including GSTP1 Ile105Val, GPX1 Pro198Leu, and SOD2 Ala16Val, modulate both baseline ROS generation and therapeutic response to antioxidant supplementation, with SOD2 Val/Val carriers showing superior clinical outcomes following combination antioxidant therapy [185].

### 8.3. Nitric Oxide Synthase Pathway Genes

Nitric oxide (NO) plays a paradoxical role in male reproduction, essential at physiological levels yet destructive when dysregulated. Produced by the NO synthase (NOS) family, NO participates in sperm capacitation, motility modulation, and acrosome reaction [186]. However, excessive generation leads to nitrosative stress, forming peroxynitrite and other reactive nitrogen species (RNS) that trigger oxidative DNA damage, protein nitration, and lipid peroxidation. Genetic polymorphisms within NOS pathway genes decisively influence this balance, positioning them as key regulators of the redox–genomic axis [187].

Three NOS isoforms, neuronal (nNOS, NOS1), inducible (iNOS, NOS2), and endothelial (eNOS, NOS3), are expressed in the testis, epididymis, and spermatozoa [188]. Among them, NOS3 polymorphisms are most intensively investigated. The Glu298Asp variant (rs1799983) alters enzyme conformation, reducing stability and enhancing proteolytic cleavage [189]. Carriers of the Asp allele exhibit lower seminal NO concentrations and impaired sperm motility, reflecting deficient vasodilation and suboptimal oxygenation within testicular microcirculation [190]. Conversely, in some contexts, this variant associates with elevated nitrosative stress, implying tissue-specific regulatory complexity [191]. Another NOS3 polymorphism, a 27 bp variable number tandem repeat (VNTR) in intron 4, modulates mRNA splicing and enzyme activity [192]. The ‘4a’ allele, associated with reduced NO bioavailability, has been linked to oligozoospermia and idiopathic infertility across multiple populations [193].

In the NOS2 gene, promoter polymorphisms such as –954G>C and C150T influence inducible enzyme expression [194]. The high-expressor genotypes generate excessive Nitric oxide during inflammation, leading to oxidative-nitrosative crosstalk that damages sperm DNA and disrupts chromatin condensation [195]. These effects are especially pronounced in conditions with testicular inflammation or infection, where macrophage activation sustains prolonged iNOS activity [186]. Downstream of NO synthesis, GTP cyclohydrolase 1 (GCH1) and arginase (ARG2) genes regulate substrate and cofactor availability [196]. GCH1 polymorphisms impair tetrahydrobiopterin (BH4) synthesis, a crucial NOS cofactor, resulting in ‘NOS uncoupling’, a pathological state in which NOS generates superoxide instead of NO, thereby intensifying OS [197]. Similarly, ARG2 variants influencing arginine metabolism can tilt the balance toward ROS production [178]. Clinically, NOS polymorphisms contribute to diverse redox–genomic endotypes. NOS3 low-activity variants correspond to reduced seminal plasma NO and lower sperm motility, whereas NOS2 high-activity alleles correlate with increased 8-OHdG and SDF [198]. Combining NOS genotyping with ORP or nitrotyrosine assays could stratify men into nitrosative versus OS–dominant phenotypes.

Therapeutic implications are emerging. L-arginine and BH4 supplementation may benefit carriers of NOS3 loss-of-function variants by restoring endothelial NO production [199], while targeted antioxidants like melatonin or tempol can neutralize peroxynitrite in high-NO genotypes. Precision in such interventions depends on identifying genotype-phenotype correlations across populations, a research direction gaining traction in redox medicine. Thus, NOS pathway genes exemplify how subtle genetic variation converts a physiological signaling system into a genotoxic stressor. This latter study reinforces the central thesis of redox–genomic crosstalk: that the fate of sperm genome is determined not only by oxidants themselves but also by the inherited blueprints governing how those oxidants are made and managed.

### 8.4. Genotype and Ancestry-Specific Cut-Offs

Accumulating evidence demonstrates substantial ancestry-related variation in DNA repair and antioxidant gene polymorphism frequencies, necessitating population-specific reference ranges for OS biomarkers and personalized treatment thresholds. The OGG1 Ser326Cys variant exhibits allelic frequency differences across East Asian, European populations, Turkish, and Middle Eastern populations [200,201]. These disparities translate to differential baseline SDF susceptibility. Pharmacogenomic studies reveal ancestry-dependent treatment responses. Russian men carrying different GSTP1 allele polymorphism demonstrated different antioxidant capacity, with a projected different response to various antioxidants [202].

More genomic studies are needed to investigate allele frequencies of OS related genes among different ethnic populations. Implementation of genotype-guided thresholds in clinical practice could show promising outcomes. Integrating genetic ancestry and individual genotype data into diagnostic algorithms and treatment decision-making for OS-mediated male infertility will optimize the use of therapeutic strategies including antioxidant therapy and ART.

## 9. Evidence to Outcomes: Natural Conception and ART

### 9.1. Predictive Value for Natural Fertility

The prognostic value of SDF and oxidative biomarkers in predicting spontaneous conception has been explored across several observational studies and meta-analyses. However, results remain inconsistent may be due to methodological differences and the scarcity of well-designed prospective data [79,203]. In a meta-analysis involving 30 studies focused on ART, Cissen et al. (2016) reported that SDF testing showed moderate sensitivity but low specificity for predicting ongoing pregnancy, with limited relevance to natural conception [204]. Similarly, Simon et al. (2016), in a broader systematic review, found that increased SDF was negatively associated with natural fertility, though most studies were constrained by small sample sizes and inconsistent assay thresholds [205]. Prospective research in men attending fertility clinics indicates that higher SDF levels are linked with longer time to pregnancy, a lower likelihood of conception within one year, and a greater incidence of unexplained or idiopathic infertility, even after adjustment for standard semen parameters. Nonetheless, interpretation of these associations is limited by confounding factors such as female reproductive health, lifestyle influences, and the lack of standardized cut-offs for defining elevated SDF. Overall, current evidence suggests that SDF and redox imbalance contribute additional prognostic insight in cases of male infertility, but they are not yet validated as independent predictors of natural conception outcomes.

### 9.2. Impact on ART Outcomes

The predictive power of SDF and redox biomarkers for ART success is rigorously examined, though results remain partly conflicting. Meta-analysis by Cissen et al. reported associations of higher SDF with reduced fertilization rates, lower embryo quality, poorer pregnancy rates, and increased miscarriage rates (IVF or ICSI), but acknowledged low specificity and methodological heterogeneity [204]. Subsequent meta-analyses and systematic reviews support a specific trend of elevated SDF in association with undesired ART outcomes, particularly for clinical pregnancy, live birth, and miscarriage endpoints. For example, Deng et al. (2019) showed adverse effects of DFI on ART success rates [206]. A 2024 study by Li et al. reported that in IVF/ICSI cohorts, patients with DFI > 30% had significantly higher miscarriage rates and lower birth weights compared to those with DFI < 15% [127]. However, some more recent investigations suggest null or weak correlations. Chen et al. (2022) did not find significant association between sperm DFI and ART outcomes in their cohort [207]. The variable assay techniques (TUNEL, Comet, SCSA, SCD), differences in DFI thresholds, and potential confounding (e.g., female age, embryo selection) moderate the interpretability of these data. Consequently, some guidelines remain cautious about recommending routine SDF testing in ART settings. Overall, the weight of evidence leans toward SDF serving as a negative prognostic biomarker in ART, especially for miscarriage and live birth endpoints, with stronger effect sizes in cases of high fragmentation (>30%) and when multiple unsuccessful cycles exist.

An emerging translational strategy involves the use of testicular sperm retrieval in men presenting with high ejaculate SDF. The rationale is that sperm collected prior to epididymal transit may exhibit fewer oxidative DNA lesions and lower SDF. A recent updated meta-analysis (2023) reported that testicular sperm show significantly reduced SDF compared with ejaculated sperm. Moreover, the use of testicular sperm for ICSI was associated with higher clinical pregnancy and live birth rates, as well as a lower incidence of miscarriage in couples affected by elevated SDF [208]. Similarly, Esteves et al. (2017) showed improved outcomes in ICSI compared with ejaculated-ICSI in high SDF patients [209]. In a systematic review by Cano-Extremera et al. (2025), pooled analysis of 13 studies confirmed that testicular sperm contributed to superior live birth rates and reduced miscarriage risk when used for ICSI in men with high ejaculated SDF; while fertilization rates were similar overall, in certain subgroups (e.g., oligozoospermic men) ejaculated sperm had better fertilization performance [210]. Mechanistically, it is proposed that post-testicular oxidative damage accumulates during epididymal transit, interaction with seminal plasma, or ejaculation; thus, retrieving sperm directly from the testis may reduce SDF by avoiding these stages. Nonetheless, several limitations persist, including the invasiveness of surgical retrieval, potential risk of testicular injury, and the absence of full maturational epigenetic remodeling typically acquired during epididymal passage. Furthermore, some studies have found no significant difference in ART outcomes between testicular and ejaculated sperm, particularly in cases with lower baseline SDF [211]. Therefore, the use of testicular sperm appears most advantageous in carefully selected patients who continue to exhibit high SDF despite corrective interventions, rather than as a generalized approach.

### 9.3. Safety and Offspring Health Considerations

Beyond achieving pregnancy, an essential concern is whether redox-induced SDF or epigenetic dysregulation translate into adverse outcomes in offspring. Some emerging evidence suggests associations of high paternal SDF with higher miscarriage, lower birth weight, and possibly increased risk of de novo mutations or epigenetic disorders. For example, Li et al. (2024) found a positive correlation between miscarriage rates and sperm DFI and a negative correlation between DFI and birth weight in ART offspring cohorts, though no significant differences were observed in stillbirth or major birth defects [127]. These findings suggest a possible transgenerational influence of redox–genomic disturbances, although a direct causal relationship has yet to be established. Moreover, the epigenetic modifications induced by OS, aberrant methylation, histone retention anomalies, and dysregulated non-coding RNAs, have theoretical risks for imprinting errors or altered developmental gene expression in the embryo, but robust human longitudinal data remain sparse. Some animal and in vitro models confirm that paternal oxidative insults can provoke epimutations and phenotypic changes in progeny, but translation to human risk is still speculative. From an ART safety perspective, the use of testicular sperm raises additional considerations. It has been suggested that bypassing epididymal maturation could influence epigenetic programming; however, current evidence does not demonstrate an increased risk of congenital or developmental anomalies in children conceived through testicular ICSI. Nevertheless, long-term follow-up data remain limited. Thus, evidence suggests that elevated SDF and oxidative disequilibrium may negatively influence early developmental and perinatal outcomes, though the absolute risk to offspring remains relatively low and insufficiently defined. From an ethical standpoint, efforts should focus on minimizing paternal OS through lifestyle optimization, nutraceutical support, and targeted redox therapies alongside careful sperm selection. Continued prospective monitoring of ART-conceived offspring within defined redox–genomic risk categories is warranted to clarify long-term implications.

Cumulative evidence indicates that elevated SDF and redox imbalance adversely affect both natural conception and ART outcomes, particularly regarding miscarriage and live birth rates. Testicular sperm use in men with high SDF shows clinical promise, yet findings must be interpreted cautiously given methodological variability, potential confounders, and scarce long-term safety data for offspring. Future well-designed prospective studies integrating redox–genomic profiling and longitudinal follow-up are essential to establish this mechanistic framework as a safe and evidence-based component of reproductive practice.

## 10. Future Directions and Trial Blueprints

The next decade in male infertility research must transition from associative observations to mechanistically driven, phenotype-stratified clinical trials. Multi-omics integration, combining redoxomics, genomics, and epigenomics, can delineate precise endotypes of oxidative and genomic dysfunction, enabling predictive diagnostics rather than retrospective explanations. Randomized controlled trials should adopt redox–genomic inclusion criteria, stratifying participants by OS, SDF index, or protamine deficiency to identify genuine responders to antioxidant or mitochondrial therapies. Such designs would refine outcome metrics beyond semen improvement, prioritizing live birth, embryo competence, and miscarriage reduction. Parallel efforts must establish region-specific reference intervals for oxidative and epigenetic biomarkers, recognizing the influence of ethnicity, diet, and climate on seminal redox physiology. Longitudinal ‘omics’ cohorts are also needed to map how transient oxidative insults translate into persistent epigenetic scars across spermatogenic cycles. Finally, open-science calibration frameworks, shared repositories for assay protocols, redox thresholds, and data normalization, will ensure reproducibility across laboratories and accelerate translation into precision andrology. The future of redox–genomic medicine, therefore, lies not in more antioxidants, but in smarter trials: those that couple biological plausibility with stratified design, transforming OS from a diffuse concept into a measurable, targetable dimension of personalized male reproductive care.

## 11. Conclusions

The redox–genome–epigenome axis provides a unifying framework to explain much of the so-called idiopathic male infertility that escapes routine diagnostics. OS, once viewed as an isolated biochemical imbalance, now emerges as a regulator of chromatin integrity and epigenetic remodeling, influencing both fertilization potential and offspring health. Recognizing distinct redox–genomic endotypes enables a move toward targeted, monitorable care instead of empirical antioxidant use. Future clinical models must integrate OS, SDF, and epigenetic markers into cohesive diagnostic panels, validated across populations and assay platforms. However, the clinical translation of these approaches is currently limited by substantial challenges in standardization, including variability in OS and SDF assays, lack of consensus on epigenetic thresholds, and heterogeneity in sample processing and analytical pipelines across laboratories. Addressing these gaps will require harmonized methodological guidelines, inter-laboratory validation, and the establishment of clinically meaningful reference ranges. Standardized protocols, transparent data sharing, and phenotype-stratified trials will be crucial to transform molecular findings into precision therapies. Ultimately, managing male infertility through the lens of redox–genomic crosstalk reframes it not as a single-organ failure but as a systems disorder, where restoring redox harmony safeguards genomic fidelity, reproductive success, and the genetic wellbeing of the next generation.

## Figures and Tables

**Table 1:** Oxidative, genomic, and epigenetic interactions in male infertility: mechanisms, biomarkers, and clinical translation.

Axis of Interaction	Principal Mechanistic Drivers	Molecular Consequences	Representative Biomarkers/Assays	Clinical Manifestation/Endotype	Therapeutic and Translational Implications
**Redox Imbalance (Oxidative Axis)**	Mitochondrial electron leakage (Complex I/III); NADPH oxidase overactivity; leukocyte infiltration; environmental ROS (smoke, metals, heat)	Lipid peroxidation (MDA, 4-HNE); protein carbonylation; oxidative base lesions (8-OHdG)	ORP (MiOXSYS); TAC; SOD, GPx, CAT activity; 8-OHdG ELISA	*Redox-dominant* or *Redox-driven SDF* phenotype; often reversible with antioxidant/lifestyle correction	Lifestyle optimization; targeted antioxidants (CoQ10, carnitine, NAC); ORP-guided therapy; monitor to prevent reductive stress
**DNA Integrity (Genomic Axis)**	Oxidative strand scission; protamine deficiency; apoptotic endonuclease activation (EndoG, AIF); defective repair (OGG1, PARP1 loss)	Single-/double-strand breaks; mtDNA mutations; chromatin cross-links	SDF assays: TUNEL, SCSA, Comet, SCD; 8-OHdG quantification	*SDF-isolated* or mixed oxidative-genomic phenotype; unexplained infertility with normal semen parameters	Varicocelectomy, infection control, antioxidant or mitochondrial stabilizers; SDF-guided ART planning
**Chromatin Remodeling**	Incomplete histone–protamine transition; BRDT/CHD5/TNP/PRM mutations; cysteine oxidation in protamines	Excess histone retention; disulfide bridge loss; open chromatin susceptibility	Protamine 1/2 ratio; Aniline blue staining; ChIP-seq for H3K4me3/H3K9me2	*Chromatin/epigenetic dysfunction with redox overlay*	Epigenetic-safe antioxidants (melatonin); sperm chromatin maturity testing before ART
**Epigenetic Dysregulation**	ROS-induced methyl-cytosine oxidation; TET dysfunction; altered histone acetylation; disturbed small RNA cargo	Global hypomethylation; imprinting errors (*H19*, *MEST*); dysregulated miRNA/piRNA	Bisulfite-seq; methylation arrays; small RNA-seq	*Epigenetic-dominant* phenotype; recurrent miscarriage or ART failure despite normal semen	Nutritional-epigenetic modulation (folate, B12, SAMe); avoidance of environmental oxidants; selection of epigenetically stable sperm for ART
**Genetic Modifiers of Redox Response**	SNPs in *SOD2 (Val16Ala)*, *GPx1 (Pro198Leu)*, *CAT (–262C>T)*, *GST nulls*, *NOS3 (Glu298Asp)*	Altered enzymatic kinetics; NO–ROS imbalance; increased susceptibility to oxidative injury	Genotyping (PCR/NGS panels); correlation with ORP and SDF	Variable expressivity across ethnic cohorts; defines heritable *redox–genomic endotype*	Genotype-guided antioxidant or NO-modulating therapy; population-specific reference intervals

4-HNE, 4-Hydroxynonenal; 8-OHdG, 8-Hydroxy-2′-deoxyguanosine; AIF, Apoptosis-Inducing Factor; BRDT, Bromodomain Testis-Specific Protein; CAT, Catalase; CHD5, Chromodomain Helicase DNA-Binding Protein 5; ChIP-seq, Chromatin Immunoprecipitation Sequencing; CoQ10, Coenzyme Q10; EndoG, Endonuclease G; GPx, Glutathione Peroxidase; GPx1, Glutathione Peroxidase 1; GST, Glutathione S-Transferase; H3K4me3, Histone H3 Lysine 4 Trimethylation; H3K9me2, Histone H3 Lysine 9 Dimethylation; H19, Imprinted Gene H19; MDA, Malondialdehyde; MEST, Mesoderm-Specific Transcript (Imprinted Gene); miRNA, Micro-Ribonucleic Acid; mtDNA, Mitochondrial Deoxyribonucleic Acid; NAC, N-Acetylcysteine; NADPH, Nicotinamide Adenine Dinucleotide Phosphate (reduced form); NGS, Next-Generation Sequencing; NOS3, Nitric Oxide Synthase 3 (Endothelial NOS); OGG1, 8-Oxoguanine DNA Glycosylase 1; ORP, oxidation–reduction Potential; PARP1, Poly(ADP-Ribose) Polymerase 1; piRNA, PIWI-Interacting Ribonucleic Acid; PRM, Protamine; SAMe, S-Adenosyl-L-Methionine; SCD, Sperm Chromatin Dispersion; SCSA, Sperm Chromatin Structure Assay; SDF, Sperm DNA Fragmentation; SNP, Single Nucleotide Polymorphism; SOD2, Superoxide Dismutase 2 (Manganese Dependent); TAC, Total Antioxidant Capacity; TET, Ten-Eleven Translocation Dioxygenase; TNP, Transition Protein; TUNEL, Terminal Deoxynucleotidyl Transferase dUTP Nick-End Labeling. Phenotypes have been indicated in *italics* in the ‘Endotype’ column.

## Data Availability

No new data were created or analyzed in this study. Data sharing is not applicable to this article.

## Abbreviations

The following abbreviations are used in this manuscript:

4-HNE	4-hydroxynonenal
5-hmC	5-hydroxymethylcytosine
8-OHdG	8-hydroxy-2′-deoxyguanosine
AB	Aniline blue
AGEs	Glycation end products
AIF	Apoptosis-inducing factor
ARG	Arginase
ART	Assisted reproductive technologies
BER	Base excision repair
BRDT	Bromodomain Testis-specific protein
CHD5	Chromodomain Helicase DNA-binding protein 5
CMA3	Chromomycin A3
DFI	Sperm DNA fragmentation index
DNMT	DNA (cytosine-5)-methyltransferase
DSBs	Double-strand breaks
GATPase	Adenosine Triphosphatase
GCH1	GTP cyclohydrolase 1
GSH	Glutathione
GSTM1	Glutathione S-transferase Mu 1
GSTT1	Glutathione S-transferase Theta 1
H19/IGF2	H19/Insulin-like Growth Factor 2
HATs	Histone acetyltransferases
HDACs	Histone deacetylases
HR	Homologous recombination
IL-6	Interleukin-6
MEST	Mesoderm Specific Transcript
Msr	Methionine sulfoxide reductase
miRNAs	MicroRNAs
MTHFR	Methylenetetrahydrofolate reductase
NAP1L4	Nucleosome Assembly Protein 1-like 4
NADP	Nicotinamide Adenine Dinucleotide Phosphate
NER	Nucleotide excision repair
NO	Nitric oxide
OGG1	8-oxoguanine DNA glycosylase 1
ORP	Oxidation-reduction potential
PARP1	Poly (ADP-ribose) polymerase 1
PRDXs	Peroxiredoxins
PRM	Protamines
PTMs	Post-translational histone modifications
Px	Glutathione peroxidase
ROS	Reactive oxygen species
SAM	S-adenosylmethionine
SDF	Sperm DNA fragmentation
SNRPN	Small Nuclear Ribonucleoprotein Polypeptide N
SOD2	Superoxide Dismutase 2
SSBs	Single-strand breaks
TET enzymes	Ten-Eleven Translocation enzymes
TNF-α	Tumor Necrosis Factor-alpha
TNP	Transition proteins
TUNEL	Terminal deoxynucleotidyl transferase dUTP nick end labeling
VNTR	Variable number tandem repeat
piRNAs	PIWI-interacting RNAs
tsRNAs	Transfer RNA-derived small RNAs
